# Promoting a sense of belonging, engagement, and collegiality to reduce burnout: a mixed methods study among undergraduate medical students in a non-Western, Asian context

**DOI:** 10.1186/s12909-022-03380-0

**Published:** 2022-04-28

**Authors:** Pongtong Puranitee, Winitra Kaewpila, Sylvia Heeneman, Walther N. K. A. van Mook, Jamiu O. Busari

**Affiliations:** 1 Department of Pediatrics, Faculty of Medicine, Ramathibodi Hospital, Mahidol University, 270 Rama VI Road, Rajthevi, Bangkok, 10400 Thailand; 2 Faculty of Medicine, The ChakriNaruebodindra Medical Institute, Mahidol University, 111 Tambon Bang Pla, Amphoe Bang Phli, 10540 Chang Wat Samutprakan, Thailand; 3 Department of Pathology, School of Health Professions Education, Faculty of Health, Medicine & Life Sciences (FHML), Maastricht University (UM), P.O. Box 616, 6200 MD, Maastricht, The Netherlands; 4 Department of Intensive care medicine, and Academy for Postgraduate Medical Training, Maastricht University Medical Center+, 6202 AZ, Maastricht, The Netherlands; 5 School of Health Professions Education, Faculty of Health, Medicine & Life Sciences (FHML), Maastricht University (UM), P.O. Box 616, 6200 MD, Maastricht, The Netherlands; 6 Department of Educational Development & Research, Faculty of Health, Medicine & Life Sciences (FHML), Maastricht University (UM), P.O. Box 616, 6200 MD, Maastricht, The Netherlands; 7 Department of Pediatrics and HOH Academy, Horacio Oduber Hospital, Dr. Horacio E. Oduber Boulevard #1, Oranjestad, Aruba

**Keywords:** burnout, medical education, professional health education

## Abstract

**Background:**

Burnout is a psychological condition induced by work-related chronic interpersonal stressors. Interventions creating a sense of belonging and collegiality have been proposed as approaches for alleviating burnout. The current study aimed to: (1) explore the relationships between burnout, sense of belonging (relatedness with others), and work engagement; and (2) identify the key elements perceived by undergraduate medical students as positively contributing to collegiality, engagement, and a sense of belonging, in an undergraduate medical training setting.

**Methods:**

An exploratory sequential mixed-methods design using questionnaires and semi-structured individual interviews collected quantitative and qualitative data among undergraduate medical students at Mahidol University, Thailand. The Maslach Burnout Inventory-Student Survey questionnaire was used to measure burnout. The Basic Psychological Need Satisfaction at Work Scale (BPNSS-21) and the Utrecht Work Engagement Scale-Student Version (UWESS-9) measured students’ basic psychological needs satisfaction at work and work engagement, respectively. Descriptive statistical analysis and confirmatory factor analysis were performed on BPNSS-21 and UWESS-9 data. Spearman’s correlation coefficient was used to identify the correlation between burnout and other factors. Twenty undergraduate medical students participated in the qualitative study. Qualitative analysis was conducted iteratively using constant comparison and the standard principles of primary, secondary, and tertiary coding for thematic analysis.

**Results:**

Thai versions of the BPNSS-21 and UWESS-9 showed an acceptable fit for the Thai cultural context. Burnout had significant weak inverse associations with engagement (r = − 0.39, *p* < 0.005) and basic psychological needs satisfaction (r = − 0.37, *p* < 0.005). Sense of belonging had a significant weak inverse relationship with burnout (r = − 0.25, *p* < 0.005). The main themes emerging from qualitative analysis were relevant tasks and learning activities, safety in the learning environment, peer interaction, program design factors, dynamics of collegiality while progressing through medical school, and personal stance and social skills.

**Conclusions:**

Sense of belonging, engagement, and collegiality were related to burnout. The key features for promoting collegiality, the sense of belonging, and engagement were relevant tasks and learning activities, safety in the learning environment, peer interaction, program design factors, dynamics of collegiality while progressing through medical school, and personal stance and social skills.

**Supplementary Information:**

The online version contains supplementary material available at 10.1186/s12909-022-03380-0.

## Background

In 1993, Maslach defined burnout as a psychological condition in response to chronic interpersonal stressors related to work [1]. However, in 2019, the World Health Organization re-defined burnout from a health perspective as a syndrome resulting from chronic and poorly managed workplace stress that is characterized by 1) high emotional exhaustion or feelings of energy depletion, 2) high depersonalization or cynicism related to one’s job, and 3) a lack of personal accomplishment or perception of low professional efficacy [2].

Previous studies have reported that the prevalence of burnout among medical students ranges between 45 and 71% [3]. Although many of these studies have been conducted in Western countries, those originating in non-Western contexts indicate that burnout is a cross-cultural problem [4]. For example, among undergraduate medical students in Thailand, the prevalence of burnout in three domains (high emotional exhaustion, high depersonalization, and low professional efficacy) combined was 28.4% [5]. In one study, Srikam et al. [6] evaluated the level of burnout among residents in Thailand. They reported that 38.8, 52.8, and 40% of the residents experienced emotional exhaustion, depersonalization, and reduced personal accomplishment.

Work engagement is a positive, fulfilling, work-related state of mind, and is considered to be a positive factor for mitigating against burnout. Thus, interventions to promote work engagement may have preventive value against burnout. Work engagement is characterized by vigor, dedication, and work absorption [7–11] and is a component of the domain of the resources in the Job Demands-Resources Model (JD-R). In both Western cultures and Asian cultures, work engagement is inversely correlated with burnout [12, 13].

Resilience is a crucial factor in promoting work engagement [14, 15]. In 2016, McKenna et al. defined resilience as the ability to bounce back when a person encounters adversity. Social resilience is the ability of a group to tolerate stress adaptively through mutual trust and bonding among its members [16]. Hence, resilience has been proposed as a critical factor in promoting well-being in the workplace [17, 18]. Social resilience can result from connecting members in the workplace with each other, which can also described be described as collegiality. According to Maslow’s hierarchy of needs, collegiality can fulfill the need for relatedness (belongingness). Belongingness refers to the desire to have a sense of belonging, and entails feeling accepted and valued by others [19, 20]. Alongside resilience, a sense of belonging is also considered to be crucial for promoting work engagement. A sense of belonging occurs when a person is involved in a system or environment in which they perceive themselves to be integral, and comprises (1) the perception of being valued for involvement, feeling accepted, and needed, and (2) a perceived fit between their individual characteristics and the system or environment [21]. In a study among Korean pharmacy students [22], the need for relatedness in psychological needs satisfaction was inversely related to burnout. Therefore, building social connections among colleagues and friends in relation to burnout may be a promising solution for alleviating burnout.

Hence, novel or alternative interventions that create a sense of belonging (being valued and perceived fit) and collegiality have been proposed as approaches for overcoming the disconnection between peers and other professionals in the clinical workplace. These interventions are expected to increase work engagement both in the clinical and pre-clinical setting, and decrease the incidence of burnout. In the Thai undergraduate medical curriculum, the learning environments differ between the clinical years (years 4–6) and the pre-clinical years (years 1–3). In the clinical years, undergraduate medical students are part of smaller groups of 10–25 students rotating in different clinical departments. Their learning environment is more authentic, including working in patients’ care teams, which involves interactions with patients, nurses, teachers, senior students, residents, and fellows. In contrast, in the pre-clinical years, students are either part of the full year group (e.g., in a lecture hall) or part of a smaller group of 30 students (e.g., in classroom or laboratory teaching). Thus, for pre-clinical students, social interactions mostly occur among peers and some teachers.

However, key factors that promote a sense of belonging (being valued and perceived fit) and collegiality related to alleviating burnout are still poorly understood, especially from a non-Western, Asian medical students’ perspective. Many non-Western medical schools, including those in Asia, have adapted medical curricula from Western medical education frameworks, because social and cultural needs and resources are fundamentally different in Western medical education frameworks, including interactions between peers and staff, societal expectations, motivations, and interests of the faculty staff [23, 24]. In addition, because Asian cultures are generally collectivist, the group’s needs and goals are typically valued above the individual’s desires. Thus, long-term relationships with group members and supporting the group are crucial for cultural values in collectivist cultural contexts [25]. Exploring these aspects among undergraduate medical students is thus essential to guide the design of new interventions to alleviate burnout in medical schools in non-Western, Asian contexts.

To investigate this issue, we examined the following research questions:

1. To what extent is burnout related to a sense of belonging (relatedness with others) and work engagement for undergraduate medical students?

2. What are the key elements that undergraduate medical students perceive as positively or negatively contributing to promoting collegiality, engagement, and a sense of belonging?

## Methods

An exploratory sequential mixed-methods design was applied to collect quantitative and qualitative data, using questionnaires and semi-structured individual interviews, respectively. This study was approved by the Ethics Committee of the Faculty of Medicine of Ramathibodi Hospital, Mahidol University.

### Quantitative component

#### Participants and setting

Medical students in years 1–6 at Ramathibodi Hospital, Mahidol University, Bangkok, Thailand, were invited by an education staff member after a plenary lecture to participate in the quantitative component of this study in the last semester of the 2020 academic year. Students that did not want to participate were welcome to leave. The questionnaires were then distributed in a written format. Students handed in completed questionnaires at the desk in front of the lecture hall, ensuring that data were collected confidentially. The research team could not track the data back to individual participants, and only the primary researcher had access to the data. A total of 1160 medical students provided informed consent for data collection. Each participant was able to withdraw from the study at any time, and no incentive was provided for participation.

#### Questionnaires

Three questionnaires were used, as follows:

1. The Maslach Burnout Inventory-Student Survey Thai version (MBI-SS), a 15-item questionnaire rated using a seven-point Likert scale, with three factors: high emotional exhaustion, high depersonalization, and low professional efficacy [5, 7, 8, 26].

2. Basic Psychological Need Satisfaction at Work Scale (BPNSS-21), a 21-item questionnaire, rated with a seven-point Likert scale ranging from “not at all true” to “very true,” and three factors: autonomy, competence, and relatedness [27–30].

3. Utrecht Work Engagement Scale-Student version (UWESS-9), a nine-item questionnaire, using a seven-point Likert scale with three factors: vigor, dedication, absorption [31–36].

The BPNSS-21 and UWESS-9 were translated, and their psychometric properties were determined for the Thai context.

#### Analysis

A descriptive statistical analysis was performed to identify participants’ burnout prevalence, basic psychological need satisfaction level, and engagement level. The psychometric properties and confirmatory factor analysis of the BPNSS-21 and UWESS-9 were examined. Psychometric sensitivity evaluation was conducted by measuring central tendency and shape. Items with skew (Sk) and kurtosis (ku) above three, in absolute values, were interpreted as sensitivity problems. The fit indices used for confirmatory factor analysis were the chi-square and degrees of freedom ratio (χ^2^/df), standardized root mean squared error (SRMR), comparative fit index (CFI), Tucker–Lewis index (TLI), and the root mean square error of approximation (RMSEA) (acceptable fit index values are provided in the Appendix). In addition, confirmatory factor analysis and Spearman’s correlation coefficient were performed using AMOS® version 18.0 and SPSS Statistics for Windows version x.0 (SPSS Inc., Chicago, IL, USA) to identify correlations between the sense of belonging, burnout, engagement, and other factors.

### Qualitative component

#### Participants

In the second part of the study, a purposeful sample of deviant cases from each year was selected. Students with the highest and lowest levels of burnout (in MBI questionnaire scores) were considered as deviant cases. These students were invited to participate in interviews via telephone calls.

#### Interviews

Semi-structured interviews were used to refine and explore factors that potentially influenced respondents’ perspectives regarding the sense of belongingness, engagement, and collegiality, determined on the basis of basic psychological needs satisfaction and engagement literature [7–11, 19, 20, 37]. Because these are sensitive topics, an individual interview approach was used. The interviews were conducted by a researcher with qualitative interview experience using a semi-structured interview guide to explore medical students’ experiences influencing their sense of belonging and collegiality in the clinical workplace or medical school. Particular attention was paid to factors that promote or inhibit the sense of belonging and collegiality among medical students. The interviews took place on site in a private room, and were digitally recorded without labeling of participants or any identifiable information.

#### Analysis

The qualitative analysis was performed according to the six-step process of thematic analysis [38]: familiarization with the data, generating initial codes, searching for themes, reviewing themes, defining and naming themes, and producing a report by applying the generally accepted principles of open coding, axial coding, and selective coding to the data [38–40]. The data analysis was conducted iteratively while data collection was still in progress to explore new topics and newly mentioned aspects in later interviews. The transcripts from the interviews were analyzed independently by two researchers (PP and WK). In cases of disagreement in interpretation, the authors discussed the results until consensus was reached. The two researchers then independently familiarized themselves with the data, generated initial codes, and reflexively considered the codes together. The preliminary coding scheme was then organized and discussed between the two researchers during the interview process until theoretical data sufficiency, or the point at which no more new information or concepts were added was reached. Searching, reviewing, defining, and naming themes was then performed iteratively, and the authors again discussed themes until agreement was reached. The final report was carefully translated into English.

#### Reflexivity

In consideration of our individual roles as researchers and the potential influence introduced in collecting, analyzing and interpreting the data, we worked as a multidisciplinary research team to mitigate the influence of positionality. PP was the lead researcher and led the data collection. PP has a background in health professions education science and is a medical teacher, but was not involved in teaching or assessing the students during this study period. Therefore, the power differential in the relationship between teacher and student was limited. WK served as co-researcher and is a medical teacher who also has a background in health professions education sciences. WK had no direct contact with the participants during the data collection period. SH has a biomedical PhD with subsequent training and experience in educational research. JB (also an educationalist) and WvM both have PhDs in medical education. Each of these researchers (SH, WvM, JB) provided independent external medical education and research perspectives on the data and its interpretation. They reviewed examples and counter-examples, supported the thematic analysis, and participated in the scientific writing process to prevent tunnel vision or confirmation distortion. In addition to medical education expertise, WvM and JB are also active physicians, enabling them to consider the results of the study in the context of contemporary clinical healthcare.

## Results

### Quantitative component

#### Psychometric properties of the BPNSS-21 (Thai version)

##### Reliability

Forward-backward translation of the BPNSS-21 indicated a strong degree of agreement, with a kappa value of 0.87. The internal consistency of the questionnaire was good, with a Cronbach’s alpha value of 0.85. The Cronbach’s alpha coefficient values for autonomy, competence, and relatedness were 0.71, 0.67, and 0.79, respectively. Mean and standard deviation are reported in Table 1.

**Table 1:** Means, standard deviations, and Cronbach’s indices of internal consistency for the BPNSS-21 and the UWESS-9 (*n* = 743)

Basic Psychological Need Satisfaction at Work Subscales	Mean	SD	Cronbach’s alpha
Autonomy (score 7–49)	29.5	5.4	0.71
Competence (score 7–42)	26.1	5.1	0.67
Relatedness (score7–56)	40.1	6.8	0.79
Overall			0.85
**UWESS-9**			
Vigor (score 0–18)	9.6	3.8	0.83
Dedication (score 0–18)	10.4	3.7	0.80
Absorption (score 0–18)	9.3	4.1	0.81
Overall			0.93

##### Confirmatory factor analysis

The confirmatory factor analysis demonstrated acceptable fit indices of the BPNSS (Thai version), which were χ^2^/df = 5.32, CFI = 0.85, TLI = 0.79, SRMR = 0.078 and RMSEA = 0.078 [41, 42]. The path diagram with standard factor loadings of the 21-item Thai version of the Basic Psychological Needs Satisfaction at Work exhibited a sufficient fit with the original factor structure (Appendix: Table 1, Fig. 1 and Table 2).

#### The Psychometric properties of the UWESS-9 (Thai version)

##### Reliability

Forward-backward translation of the UWESS-9 revealed a kappa of 0.84, corresponding to a substantial level of agreement. In addition, the internal consistency of the questionnaire was good, with a Cronbach’s alpha value of 0.93. Mean, standard deviation, and Cronbach’s indices of internal consistency of subscales are reported in Table 1.

##### Confirmatory factor analysis

Confirmatory factor analysis demonstrated acceptable fit indices of the UWESS-9 Thai version by most of the fit indices: CFI = 0.93 TLI = 0.89, and SRMR = 0.05. χ^2^/df and RMSEA showed a moderate fit of the model in the Thai context. Further adjustment of some items in the questionnaire might result in a better fit (Appendix, Table 2).

The path diagram with standard factor loadings of the Thai version of the UWESS-9 exhibited a sufficient fit with the original factor structure (Appendix: Table 2, Table 3 and Fig. 2).

### Demographic characteristics of the participants

Of the invited 1160 students, 763 (response rate 65.8% [49.5% male]) participated in the study. The participants’ demographic characteristics are shown in Tables 2 and 3.

**Table 2:** Participants’ baseline characteristics (*n* = 763)

Baseline characteristics	Total	
	n (total)	Response rate (%)
**Year**		
1st	45 (211)	21.3%
2nd	182 (207)	87.9%
3rd	119 (202)	57.5%
4th	147 (182)	80.8%
5th	178 (178)	100%
6th	92 (180)	51.1%
**Grade point average**		
2.00–2.49	16	2.1%
2.50–2.99	96	12.9%
3.00–3.49	284	38.1%
> 3.49	350	46.9%
**Sex (*n*** **= 757)**		
Male	375	49.5%
Female	382	50.5%
**Age (years)**		
Median (IQR)	21 (IQR2), (range: 19–39)

**Table 3:** Number of students with an indicator of burnout (*n* = 709)

Burnout (MBI-SS)	*n* = 709	Percentage
Overall	139	19.6%
Personal efficacy		
• High	204	28.0%
• Moderate	230	31.6%
• **Low**	**295**	**40.5%**
Emotional exhaustion		
• Low	178	24.0%
• Moderate	179	24.2%
• **High**	**384**	**51.8%**
Depersonalization		
• Low	49	6.7%
• Moderate	236	32.1%
• **High**	**451**	**61.3%**

The Thai version of the MBI-SS questionnaire was previously validated and reported to have a good fit and reliability in the Thai context [5]. After excluding missing data and extreme responses from data analysis, 709 participants remained.

### Association between engagement, basic psychological needs satisfaction, and burnout

Age, level of training, grade point average, and sex were not significantly associated with burnout level and subscales. This study found that burnout had a weak significant inverse association with engagement (r = − 0.39, *p* < 0.005) and basic psychological needs satisfaction (r = − 0.37, *p* < 0.005). Students with an indicator of burnout had lower engagement and basic psychological needs satisfaction. In addition, relatedness with other (sense of belonging), which is one of the BPNSS subscales, shows a significant weak inverse relationship with burnout (r = − 0.25, *p* < 0.005). Professional efficacy, one of the burnout subscales, also had a higher correlation with engagement (r = − 0.52, *p* < 0.005) (Table 4).

**Table 4:** Correlations between burnout, engagement and basic psychological needs satisfaction, and their subscales

Variables		Burnout (MBI-SS)			
		Overall	Low professional efficacy	High emotional exhaustion	High depersonalization
**UWESS-9**	**Overall**	−0.39	−0.52	−0.35	−0.35
	**Vigor**	−0.39	−0.47	−0.35	−0.32
	**Dedication**	− 0.36	− 0.56	− 0.32	− 0.37
	**Absorption**	− 0.41	− 0.44	− 0.30	− 0.29
**BPNSS-21**	**Overall**	− 0.37	− 0.40	− 0.38	− 0.39
	**Autonomy**	− 0.32	− 0.32	− 0.38	− 0.30
	**Competence**	− 0.38	− 0.46	− 0.38	− 0.38
	**Relatedness**	− 0.25	− 0.28	− 0.24	− 0.30

All variables, *p*-value < 0.005

Engagement and basic psychological needs satisfaction were also significantly correlated (r = 0.51, *p* < 0.05, Table 5) and between subscales. Autonomy and competence exhibited a significant moderate correlation with engagement, and a stronger correlation than that between autonomy and relatedness.

**Table 5:** Correlation between engagement and basic psychological needs satisfaction

Variables/correlation		UWESS-9				BPNSS-21			
		Vigor	Dedication	Absorption	Overall	Autonomy	Competence	Relatedness	Overall
**UWESS-9**	Vigor	1							
	Dedication	0.81	1						
	Absorption	0.79	0.80	1					
	Overall	0.93	0.93	0.93	1				
**BPNSS-21**	Autonomy	0.43	0.46	0.39	0.46	1			
	Competence	0.45	0.54	0.48	0.53	0.61	1		
	Relatedness	0.30	0.37	0.23	0.32	0.56	0.51	1	
	Overall	0.46	0.54	0.43	0.51	0.85	0.81	0.86	1

All variables, *p* value < 0.005

In conclusion, burnout exhibited a weak but significant correlation with both the sense of belonging (relatedness with others) and work engagement among Thai undergraduate medical students.

### Qualitative component

Ten undergraduate medical students from pre-clinical years (70% male) and 10 students from clinical years (90% male), with signs of burnout (nine students, 89% male) and without signs of burnout, were invited to participate in the second part of the study. The key elements that were perceived as contributing (positively and negatively) to promoting collegiality, engagement, and a sense of belonging were explored. The main themes that emerged from the analysis were relevant tasks and learning activities, safety in the learning environment, peer interaction, program design factors, dynamics of collegiality while progressing through medical school, and personal stance and social skills**.** There was no difference in the themes identified for the students with signs of burnout versus those without signs of burnout. In the following sections, these themes will be consecutively discussed in depth.

Elements in the learning environment that were reported to be necessary for a sense of belonging were tasks, safety, and teachers, which were intertwined with program design factors. Students indicated that when these elements were perceived as helpful and supportive, this led to a perception of trust and feeling competent, strengthening the sense of belonging to the medical school and institute. These factors will be consecutively discussed in the sections below.

### Relevant tasks and learning activities

How students perceived the learning activities and tasks offered as part of the curriculum was also an essential element of a good perceived fit, as well as feeling valued and engaged.

In the pre-clinical years, tasks that benefited their team and peers were perceived as meaningful for integration with the medical school community. These could also be extra-curricular tasks.

*“I felt valuable to the faculty and my team when I contributed to an open house activity. I was responsible for sharing my experience as a medical student with secondary school students and parents. My team got really nice feedback about the usefulness of my contribution.” (ID17, pre-clinical year, no indicators of burnout).*

Among students in the clinical years, tasks related to genuine patient care and authentic tasks directly benefiting patients were perceived as meaningful and increased the sense of belongingness and engagement. Participants described “meaningless tasks” as non-physician tasks, which they perceived as not being directly related to patient encounters or care, such as completion of a long record form, which was known to be discarded after patient discharge from the hospital. When students were allowed more autonomy in their clinical work, this led them to feel trusted and competent. In addition, they felt more connected to the team:

*“I detected a depressive mood in one of my patients, and the resident trusted me, and assigned me to talk with my patient. I felt that my role was meaningful and that I belonged with the team.” (ID6, pre-clinical year, no indicators of burnout).*

*“At the affiliated hospital where I was allowed to perform real physician tasks under supervision, I felt more competence and learned more.” (ID5, clinical year, exhibited indicators of burnout).*

However, it was also evident that tasks or assignments were sometimes perceived as meaningless or not constructively aligned. In such cases, suboptimal program design prevented students from having a sense of belonging. Notably, elements related to a sense of belonging were often connected to being part of the team, which was related to the perception of collegiality.

### Safety in the learning environment

Because the context differed between pre-clinical and clinical years, the key elements identified as being related to promoting collegiality and a sense of belonging were therefore consecutively discussed separately for the clinical years and pre-clinical years.

#### The clinical year learning environment

Feeling safe in the learning environment was an essential contributory element for the sense of belonging. It was particularly evident in the clinical workplace but was also reported for the pre-clinical years. In the clinical workplace, receiving constructive feedback, feeling respected and being part of the clinical team promoted the feeling of safety and sense of belonging.

*“I felt valued in the team following a situation in which a patient and his family got financial support from the institution foundation with medical care team assistance. The family appreciated the team, and also respected me. I felt capable of helping the patient and the family.” (ID9, pre-clinical year, no indicators of burnout).*

The role of the teacher was considered to be particularly important for creating a safe learning environment. In addition to their actual presence and interactions, their teaching skills were crucial in creating a sense of belonging. Teachers who were able to create meaningful tasks (as described above), exhibit genuine engagement, and use constructively aligned teaching activities and techniques such as thinking aloud, also contributed to a sense of belonging to the medical program or institute.

*“Residents asked me to think aloud and waited for my answer rather than hurrying to finish the care plan. I understood and learned a lot more and it engaged me*. *.. When a teacher taught other topics that were not related to my real patients, I disengaged. Nevertheless, if it was related to my real patients, I would be engaged.” (ID6, pre-clinical year, no indicators of burnout).*

In contrast, particularly among students in the clinical years, belittlement, a lack of interaction, and negative remarks caused students to feel a sense of disconnection regarding perceived fit and being valued, and their sense of belonging. Consequently, they felt disconnected from the team:

*“During the bedside teaching round, if the patients were not directly assigned to my responsibility, I would not care. I would step back behind my friends and let them present their cases to the teacher. They were my shield, and protected me from being belittled by teachers or residents.*. *. I wanted to detach from the patient care team when I felt insecure in my knowledge, because I knew that residents or teachers would belittle me.” (ID1, clinical year, exhibited indicators of burnout).*

#### Pre-clinical year learning environment

Although interactions with teachers in the pre-clinical years was less intense, with more plenary teaching formats, meaningful one-on-one interactions between student and teacher created a sense of belonging and feeling supported. In addition, in the pre-clinical setting, interactions with peers felt more critical for a sense of belonging to the medical program and institute.

*“In pre-clinical year extra-curricular activities, a teacher listened to my opinion and showed openness to new ideas. I felt engaged in the activities and had a sense of belonging to the medical school. Openness to new ideas is part of the culture of the faculty.” (ID17, pre-clinical year, no indicators of burnout).*

*“I felt like I belonged at my institution because I met new people with shared values who supported each other, as well as experiencing a positive learning environment in which the students shared common characteristics.” (ID11, pre-clinical year, no indicators of burnout).*

### Program design factors

Program design factors emerged as an important interface with the tasks, safety of the learning environment, and the teacher as elements in building a sense of belonging. In addition, busy or conflicting schedules were mentioned as interfering with a sense of belonging to a group of friends, the peer group, or the team at the clinical workplace.

*“I felt closer to my friends who spent more time with me in extra-curricular activities. However, they were unable to join the same activities as me, because our overcrowded curriculum did not give us enough time. .. Some days I had no collaboration or e-lecture learning all day. I could feel a distance between friends because we talked less and knew little about each other’s lives.” (ID7, pre-clinical year, having indicators of burnout).*

Although the mentoring support system was appreciated and led to a sense of belonging in some cases, the organizational structure needed to be correct and fit-for-purpose.

*“When I felt down or blue, I tried to seek mental health support at the hospital, but I couldn’t access help. The activities of the mental health group support for medical students were good, but space was very limited. I wanted to join the activities, but I wasn’t able to access them.” (ID16, pre-clinical year, no indicators of burnout).*

Maintaining or forming peer group relationships was reported to lead to perceived collegiality. Program design factors such as busy time schedules were sometimes reported to interfere with maintaining and forming peer groups, and an inability to connect with the established peer group and a lack of support could lead to a loss of perceived collegiality.

*“In year 3, I had problems with some friends in my group (from the first year), and I left my group because I felt uncomfortable hanging out with them. I tried to join other groups, but I could not catch up with their interests or conversations. It was challenging, and I had no support.” (ID16, pre-clinical year, no indicators of burnout).*

### Dynamics of collegiality while progressing through medical school

As indicated above, a central theme in feeling a sense of belonging was feeling like part of a team and appreciating peers and staff as colleagues. As perceived by medical students, the analysis showed that collegiality had a dynamic nature during the medical training program, from being a novice first-year student to being a pre-clinical then a clinical student, and when facing unexpected events. In the pre-clinical years, most of the learning activities of all students were jointly scheduled in the same department and at the same time, such as on-site lectures and small group discussions, which allowed day-to-day contact amongst peers. In addition, there were extra-curricular activities that strengthened teamwork. In the clinical years, medical students rotated to different departments or hospitals, and had to work with a new group of peers and shared responsibility for patient care or learning assignments. Students then had less contact time with their friends from pre-clinical years, but more contact time with teachers or seniors in clinical workplaces. In addition to the shift in the types of contact, increased time pressure, busy clinical settings, and increased responsibilities meant that students had less time available for non-university activities with peer groups. When peers took responsibility for each other and helped out, there was a strong sense of collegiality and belonging.

*“In a clinical clerkship, friends in my group supported each other. We reminded each other to complete medical documents, notified each other when the laboratory results of patients were reported, and communicated all the time about group work and individual assignments. Also, we went everywhere together and made sure no one was left behind or missed anything” (ID13, pre-clinical, no indicators of burnout).*

### Personal stance and social skills

Personal stance and ability to be active in teamwork were needed for practical working relationships and experiencing collegiality. A possible mismatch in expectations was not always communicated and could lead to problems in peer relationships or disengagement when a sense of belonging to the group was not perceived.

*“I came from a secondary school that emphasized self-study and group discussion, but my friends from (another) Thai secondary school preferred to read quietly with the group. It was not my style, and I thought I disturbed their study when we studied together. Later on, I studied alone or only with friends from the same secondary school.” (ID7, pre-clinical year, no indicators of burnout).*

## Discussion

The findings related to the two research questions are discussed consecutively below, in accord with the exploratory sequential mixed-method approach applied. Regarding the first research question (determining the extent to which burnout is related to the sense of belonging [relatedness with others] and work engagement among undergraduate medical students), the current results revealed a significant weak correlation between burnout and both the sense of belonging (relatedness with others) and work engagement among Thai undergraduate medical students. The results revealed that undergraduate medical students who engaged more with their learning environment and their peers had a lower risk of burnout. Recently however, a systematic review on the impact of peer support provided inconclusive evidence for the effectiveness of interventions on student well-being [43]. However, the current findings are in accord with a similar finding in a previous study [44] among PhD students in medicine, in which the frustration of the basic psychological needs of autonomy, competence, and relatedness with others constituted the essential variables that led to burnout. In conclusion, our study revealed that all subscales of basic psychological needs satisfaction, including perceiving a high level of autonomy, competence, and relatedness with others, were associated with a lower likelihood of having an indicator of burnout. This finding is in line with basic psychological need satisfaction theory, as a critical factor in achieving better well-being [45, 46]. Thus, promoting basic psychological needs satisfaction may be beneficial for preventing burnout.

This current study also revealed evidence of an inverse relationship between engagement and burnout among postgraduate medical students. Thus, those who had a higher level of engagement with a medical school or workplace were less likely to exhibit a burnout indicator, in accord with previous findings in Western and Eastern medical students [47–49]. Regarding the relationship between basic psychological need satisfaction and engagement, the quantitative component of this study revealed that medical students who had a higher level of basic psychological needs satisfaction and subscales, namely autonomy, competence, and relatedness with others, had a higher level of engagement with the medical school. In addition, students who had higher levels of autonomy and competence had higher engagement levels compared with students who had a high level of relatedness with others. This finding is in accord with the results of previous studies of university students in Malaysia [50] and medical students in Korea [51]. The evidence from this study in the Thai context is similar to those of previous studies reporting that burnout is inversely related to the fulfillment of basic psychological needs and engagement [52, 53]. These findings suggest that medical schools should design curricula that engage students and fulfill their basic psychological needs.

The second aim of this sequential mixed methods study was to clarify the key elements that contribute to promoting collegiality, engagement, and the sense of belonging, from the perspective of undergraduate medical students. The results indicated that the learning tasks and extracurricular activities relevant to medical students were crucial for engaging them. Perceived personal relevance and a meaningful connection to the individual are known to stimulate motivation and energize learning [54]. In addition, learning environments that are targeted more toward education rather than health services are reported to increase motivation and avoid a feeling of being abandoned among students [55, 56]. The current study identified several other factors that strongly affected engagement and collegiality: the psychological safety of the learning environment in both the classroom and clinical workplaces, and among peers, teachers, seniors, and other health professionals. This result is in accord with previous findings reported in the literature [57]. The learning environment is reported to be an important context, with psychosocial and material dimensions [57]. The psychosocial dimension of the framework included the individual interacting with others and social relationships with others, such as having a good community of peers and a good relationship with staff, including receiving constructive feedback, understanding the clarity of expectations, and gaining trust from patients. Students’ perception of learning environment components encompassed empathy, burnout, and quality of life [57], and students’ learning occurred when they were invited and involved in the learning environment [58]. Moreover, teacher-student relationships play a crucial role in low-stakes assessments, which stimulate self-regulated learning. Teachers could be made aware of their impact and strive for approachability and meaningful interaction, which could potentially have a positive impact on students’ perceptions of learning assessments. Therefore, medical teachers and program directors should emphasize the promotion of a positive learning environment to foster students’ learning, engagement, collegiality, and well-being [59].

In addition to the findings described above, students reported the need for a sense of social competence to build positive peer relationships and collegiality. Social awareness and relationship management skills are considered to be essential components of social competence, and have been reported to be positively associated with enhancing teamwork and leadership skills, stress management, physician wellness, and alleviating burnout [59–62]. Thus, promoting social competence among students should be emphasized to foster collegiality, as well as promoting students’ wellness and alleviating burnout.

The burnout rate found in this study was 19.6%, which was lower than that reported in a previous study (28.4%) conducted in 2016 in the same medical program. This could be the result of the renewal of student support systems and increased awareness of the problem of burnout in the institution.

Furthermore, in this study, the BPNSS-21 was translated and tested for content validity and reliability in the Thai language. The BPNSS-21 was designed to address psychological need satisfaction at work. It has been used widely [27–30], and has evolved and changed since its first use in 1992 [30]. This scale has been validated in many languages and used worldwide in family environments, workplaces, and higher education contexts [27]. The results of the current study indicated that the BPNSS-21 had good psychometric properties among Thai undergraduate medical students.

The UWESS-9 questionnaire was used to measure the level of engagement. The UWESS-9 has been adapted for use in various countries, including Finland [33], Japan [34], Portugal [35], South Africa [36], and Russia [63], and has been validated with physicians, nurses, paramedics, and medical students. In the current study, the UWESS-9 was tested for psychometric properties and showed good reliability and moderate fit with the original version in the Thai context. These two questionnaires are examples of scales that were developed in a Western context but can be generalized for use in an Eastern context. Thus, other psychological questionnaires could potentially be adapted to in Eastern context.

### Future perspectives

Our findings suggest that it is important for medical schools to explicitly establish strategies to enhance a sense of belonging, engagement, and collegiality from the first year through to the last year. From the qualitative insights of the current study, program design was an essential component to give students the opportunity to build collegiality, and to promote engagement by creating positive learning environments. Participatory strategies may be effective approaches for the design of learning environments in which multiple stakeholders, such as students, teachers, nurses, post-graduate medical students, and educational leaders are involved, combining various viewpoints [64, 65]. In addition, the design process should consider ways of enabling medical students to encounter meaningful tasks, eliminating unnecessary non-physician tasks, and matching the level of difficulty of the tasks with the level of medical students’ ability, which could promote the relevance of the learning activities for them. In addition, avoiding overcrowded curricula should be considered to provide more time and space for teamwork development and social skill development activities. Faculty development programs and resident as teacher programs should also be reconsidered to create a safe learning environment and a culture of psychological safety in medical schools. Medical schools should consider a participatory program revision to promote the sense of belonging, engagement, and collegiality. In addition, the dynamics of collegiality while progressing through medical school should be taken into account during such revision to alleviate burnout. Implementation of such a revised program and examination of its effectiveness in alleviating medical students’ burnout should be the focus of future research.

### Strengths and limitations

We believe that the current findings can be generalized to other medical schools in which the curriculum consists of both pre-clinical and clinical levels, and could be applied to enhance student well-being programs. A general limitation of this study was that the response rate per year varied between 21.3 and 100%. This relatively low response rate may have impacted the results of the quantitative data analysis. Consequently, the results may not fully represent the perceptions of first-year medical students.

## Conclusion

The current study revealed that the BPNSS-21 and UWESS-9 (Thai version) questionnaires can be used in the Thai cultural context. This result has important implications for the use of these questionnaires across cultural contexts. Second, the current findings indicated that a sense of belonging, engagement, and collegiality are important factors related to burnout. Thus, enhancing students’ autonomy, competence, relatedness, and engagement with the learning environment and peers has the potential to alleviate the problem of burnout in students. Finally, the current study identified a number of key features that could be used to promote collegiality and the sense of belonging and engagement: relevant tasks and learning activities, safety in the learning environment, peer interaction, certain program design factors, and dynamics of collegiality while progressing through medical school, as well as personal stance and social skills.

## Supplementary Information

**Additional file 1: Table 1.** Results of the confirmatory factor analysis of the Thai version of the Basic Psychological Need Satisfaction at Work (*n* = 708). **Fig. 1**. Path diagram with standard factor loadings of the 21-item Thai version of the Basic Psychological Need Satisfaction at Work (*n* = 708). **Table 2.** Standardised coefficients of the relationship between factors and items of the Thai version of the Basic Psychological Need Satisfaction at Work (*n* = 708). **Table 3.** Results of the confirmatory factor analysis of the Thai version of the Utrecht Work engagement scale-student version 9-item (*n* = 743). **Fig. 2.** Path diagram with standard factor loadings of the Thai version of the Utrecht Work engagement scale-student version 9-item (*n* = 743). **Table 4.** Standardised coefficients of the relationship between factors and items of the Thai version of the Utrecht Work engagement scale-student version 9-item (*n* = 743).

## Data Availability

For data availability, please contact Pongtong Puranitee (pongtongung@gmail.com).

## Abbreviations

BPNSS: Basic Psychological Need Satisfaction at Work Scale
CFI: Comparative fit index
GPA: Grade point average
IQR: Interquartile range
MBI-SS: Maslach Burnout Inventory-Student Survey
RMSEA: Root mean square error of approximation
SRMR: Standardized root mean square residual
TLI: Tucker Lewis Index
UWESS: Utrecht Work Engagement Scale-Student version
X2/df: Chi*-*square divided by the degrees of freedom
