# Supplementary material for: Promoting a sense of belonging, engagement, and collegiality to reduce burnout: a mixed methods study among undergraduate medical students in a non-Western, Asian context
Source: BMC Med Educ. 2022 Apr 28;22:327. doi: 10.1186/s12909-022-03380-0 (PMC9047274; doi:10.1186/s12909-022-03380-0)
Supplement: Supplementary file 1 — Additional file 1: Table 1. Results of the confirmatory factor analysis of the Thai version of the Basic Psychological Need Satisfaction at Work (n = 708). Fig. 1. Path diagram with standard factor loadings of the 21-item Thai version of the Basic Psychological Need Satisfaction at Work (n = 708). Table 2. Standardised coefficients of the relationship between factors and items of the Thai version of the Basic Psychological Need Satisfaction at Work (n = 708). Table 3. Results of the confirmatory factor analysis of the Thai version of the Utrecht Work engagement scale-student version 9-item (n = 743). Fig. 2. Path diagram with standard factor loadings of the Thai version of the Utrecht Work engagement scale-student version 9-item (n = 743). Table 4. Standardised coefficients of the relationship between factors and items of the Thai version of the Utrecht Work engagement scale-student version 9-item (n = 743). [file 12909_2022_3380_MOESM1_ESM.docx]

**Additional File 1**

**Table 1.** Results of the confirmatory factor analysis of the Thai version of the Basic Psychological Need Satisfaction at Work (n= 708)

| **Index** | **Value** | **Value indicating good fit** |
| --- | --- | --- |
| Chi-square/degrees of freedom | 5.3 | <5 |
| RMSEA | 0.078 | <0.08 |
| CFI | 0.85 | >0.90 |
| TLI | 0.79 | >0.80  >0.95 |
| SRMR | 0.078 | <0.08 |

**Figure 1**. Path diagram with standard factor loadings of the 21-item Thai version of the Basic Psychological Need Satisfaction at Work (*n*=708)


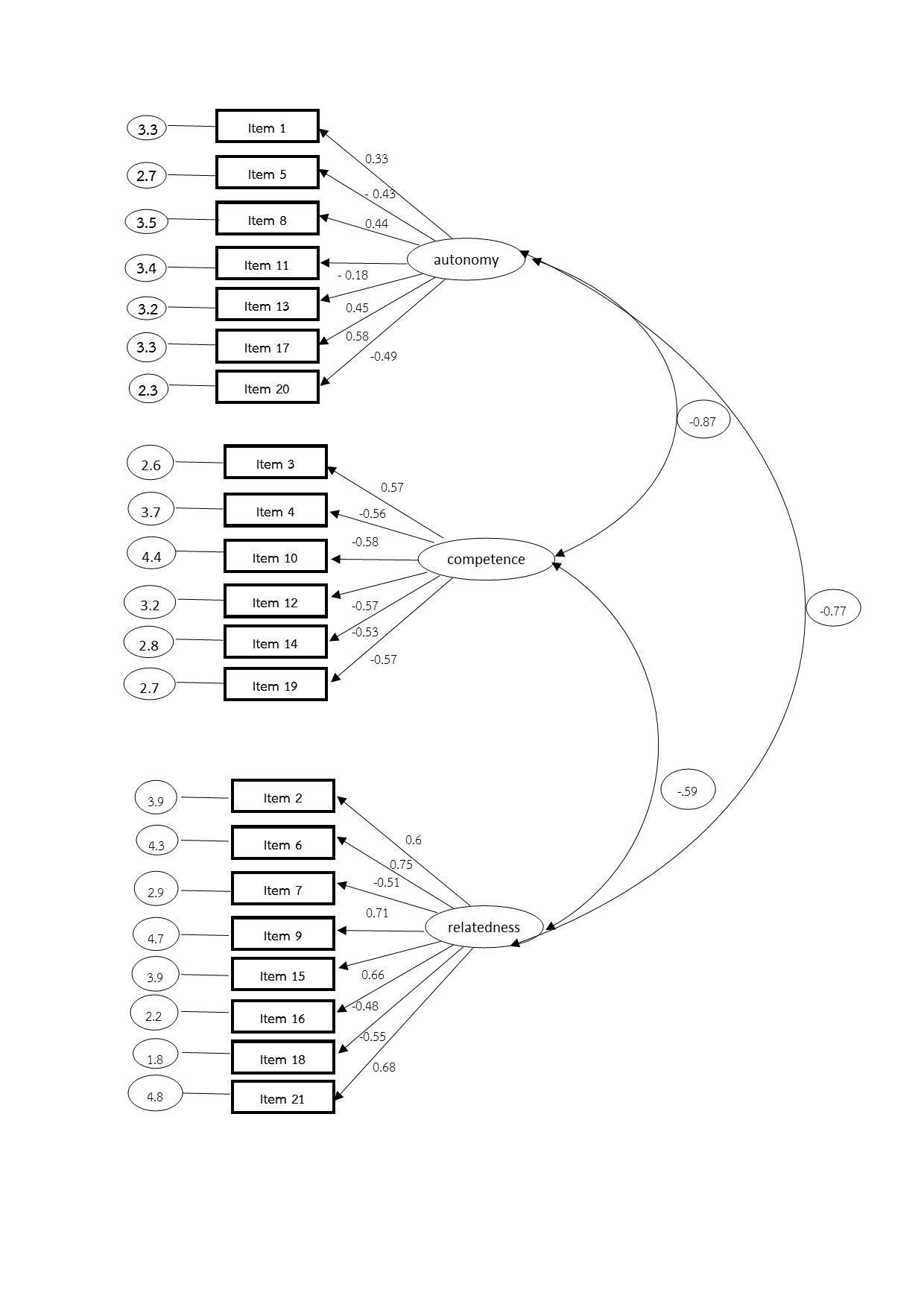


**Table 2.** Standardised coefficients of the relationship between factors and items of the Thai version of the Basic Psychological Need Satisfaction at Work **(*n*=708)**

|  | **Coefficients** | **Standard Error** | **Factor Loadings** |
| --- | --- | --- | --- |
| **Autonomy** | | | |
| item 1 | 1 | 0 | 0.333 |
| item 5 | -1.468 | 0.229 | -0.427 |
| item 8 | 1.303 | 0.189 | 0.442 |
| item 11 | -0.572 | 0.152 | -0.184 |
| item 13 | 1.327 | 0.191 | 0.452 |
| item 17 | 1.832 | 0.241 | 0.581 |
| item 20 | -1.685 | 0.235 | -0.492 |
| **Competence** | | | |
| item 3 | 1 | 0 | 0.571 |
| item 4 | -0.758 | 0.082 | -0.546 |
| item 10 | -0.799 | 0.084 | -0.577 |
| item 12 | -0.837 | 0.081 | -0.570 |
| item 14 | 0.825 | 0.089 | 0.527 |
| item 19 | 0.987 | 0.070 | 0.573 |
| **Relatedness** | | | |
| item 2 | 1 | 0 | 0.595 |
| item 6 | 1.170 | 0.083 | 0.749 |
| item 7 | -1.008 | 0.140 | -0.510 |
| item 4 | 1.134 | 0.074 | 0.711 |
| item 15 | 1.089 | 0.088 | 0.658 |
| item 16 | -0.974 | 0.111 | -0.427 |
| item 18 | -0.901 | 0.085 | -0.550 |
| item 21 | 1.049 | 0.077 | 0.684 |

**Table 3.** Results of the confirmatory factor analysis of the Thai version of the Utrecht Work engagement scale-student version 9-item (n=743)

| **Index** | **Value** | **Value indicating good fit** | **Ref** |
| --- | --- | --- | --- |
| Chi-square/degrees of freedom | 15.0 | <5 | [63] |
| RMSEA | 0.14 | <0.08 | [38] |
| CFI | 0.93 | >0.90 | [39] |
| TLI | 0.89 | >0.80  >0.95 | [63]  [39,64] |
| SRMR | 0.05 | <0.08 | [39] |

**Figure 2. Path** diagram with standard factor loadings of the Thai version of the Utrecht Work engagement scale-student version 9-item (n=743)

**
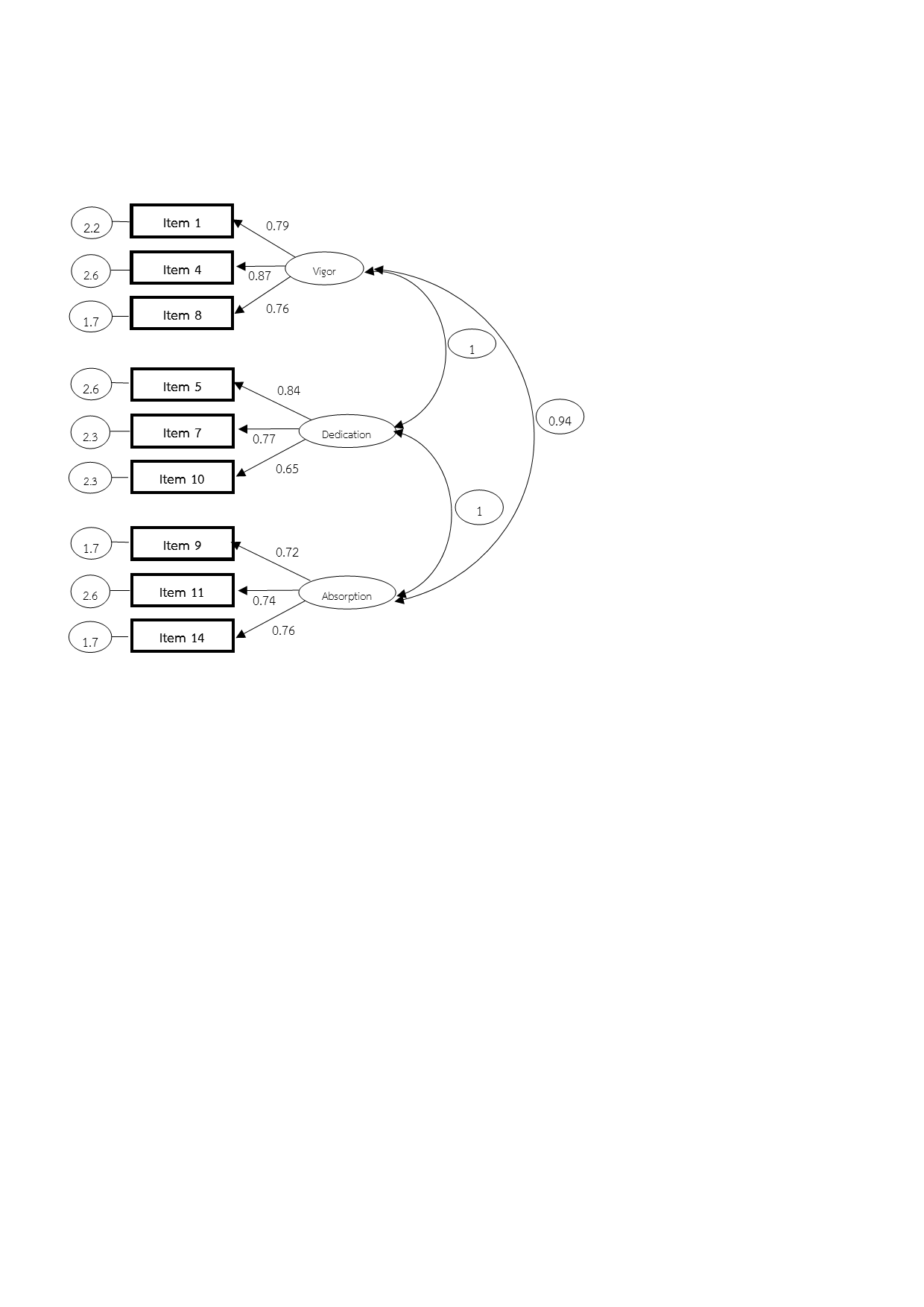
**

**Table 4.** Standardised coefficients of the relationship between factors and items of the Thai version of the Utrecht Work engagement scale-student version 9-item **(*n*=743)**

|  | **Coefficients** | Standard error | Factor loadings |
| --- | --- | --- | --- |
| **Vigor** | | | |
| item 1 | 1 | 0 | 0.788 |
| item 4 | 1.006 | 0.039 | 0.871 |
| item 8 | 1.024 | 0.047 | 0.763 |
| **Dedication** | | | |
| item 5 | 1 | 0 | 0.837 |
| item 7 | 0.986 | 0.040 | 0.768 |
| item 10 | 0.911 | 0.046 | 0.648 |
| **Absorption** | | | |
| item 9 | 1 | 0 | 0.720 |
| item 11 | 0.886 | 0.047 | 0.740 |
| item 14 | 1.045 | 0.045 | 0.762 |
